# FBXW7 as a Factor of the African American and White Breast Cancer Racial Disparity

**DOI:** 10.7759/cureus.69947

**Published:** 2024-09-22

**Authors:** Eric Pan, Haixiao Zhao

**Affiliations:** 1 Biology, DeBakey High School, Bellaire, USA; 2 Medicine, Daqing Longnan Hospital, Daqing, CHN

**Keywords:** bioinformatics tools, breast cancer, fbxw7, gene expression analysis, genetics, racial disparity, somatic mutation analysis

## Abstract

Introduction: African American women have a breast cancer mortality rate 40% higher than Caucasian women. Many contributing factors account for this racial disparity, such as socioeconomic status and the age when women give birth, but even after considering such factors, studies have found that the racial disparity persists, suggesting that genetic factors may play a crucial role in this breast cancer racial inequality.

Methods: This study utilizes the All of Us database, The Cancer Genome Atlas (TCGA), and an array of bioinformatics tools to integrate differential mutation and gene expression analyses, aiming to identify genes potentially associated with this racial disparity. Although previous studies have identified genes associated with this breast cancer racial disparity through mutation or gene expression analysis, no studies have considered both simultaneously. Ultimately, this study considers both mutation and gene expression to discover novel genes linked to this racial disparity.

Results: After mutation analysis, this study identified *FBXW7, *a gene involved in the destruction of oncogenic proteins, as being associated with this racial inequality. *FBXW7 *was the only gene that presented differences in both mutation frequency and gene expression between African Americans and Caucasians. The other four candidate genes, such as *COL12A1*, whose upregulation plays a critical role in tumor progression, may also be linked to this racial inequality.

Conclusion: By combining both mutation and gene expression analysis, this research offers a unique perspective into this issue. Furthermore, the identification of *FBXW7* provides insight into this racial disparity, which can contribute to the pursuit of more effective or personalized treatment for both Caucasian and African American breast cancer patients. Finally, the multi-level method presented could possibly apply to other racial disparities, providing a distinctive perspective that cannot be found with other methods.

## Introduction

Background

Cancer is one of the leading causes of death, and in 2024, a total of 611,720 people in the United States are expected to die of cancer, reflecting the importance of discovering more about this condition [1].

One of the characteristics of breast cancer is the presence of racial disparity and the various cancer mortality rates in different racial groups [2,3]. Breast cancer, the second leading cause of cancer-related deaths in the USA for women, is characterized by a 40% higher mortality rate in African American women than in White women [3].

Contributing factors that may account for this difference in breast cancer mortality rate include socioeconomic status and the age when women give birth [3-5]. However, after considering contributing factors such as socioeconomic status and treatment differences, studies have shown that the racial disparity persists, indicating that genetics play a major role in this racial disparity [6-8]. Previous studies have investigated possible genes that may be associated with this difference in breast cancer mortality rate by considering the expression of cancer genes [9-11] or variants in cancer genes [12,13].

Gene expression

Gene expression, the process by which the information from DNA is used to create proteins or noncoding RNA, has been an interesting avenue of research for cancer disparities, particularly between African American and White breast cancer patients. For example, a review paper by Deshmukh et al. compared the gene expression of stromal cells in vitro between African American and White women in the tumor microenvironment [14]. Overall, Deshmukh et al. identified 13 genes from various studies that were differentially expressed in African American and White breast cancer patients [14]. Tumor cells cannot survive in isolation, and as a result, they must interact with other cells in the body, including fibroblasts and immune cells [14]. The overall ecosystem that forms around a tumor is thus called the tumor microenvironment [14]. They identified 13 tumor microenvironment-related genes associated with this breast cancer racial disparity [14].

Ping et al. also compared the gene expression profiles of breast tumors between African American and European American women to identify 59 genes that were differentially expressed in African American and European American women from the Southern Community Cohort Study [9]. They hypothesized that if a certain gene was differentially expressed between the two races, then it may contribute to breast cancer racial disparity [9]. Although Ping et al. were able to identify a list of 59 genes that could differentiate between African American and European American tumors, none of the 59 genes were found to be significantly associated with survival, even after adjusting for multiple comparisons [9], demonstrating a drawback of considering only gene expression in the context of racial disparity.

To address this drawback, we implemented a multi-level method to investigate how changes in gene expression relate to survival in the context of this breast cancer racial disparity.

Gene mutations

While gene expression is a viable avenue of research, focusing on genetic mutations, a change in the DNA sequence that may contribute to cancer, is another option to identify genes associated with breast cancer racial disparity. For instance, in a literature search by Prakash et al., they reported a study that employed The Cancer Genome Atlas (TCGA) to perform somatic mutation analysis between White and African American women with breast cancer [12]. They reported numerous high prevalence genes (>5% in the TCGA dataset), including *TP53*, *PIK3CA*, and *MLL3*, that were mutated much more frequently in one race than in the other, implying that these genes may affect the difference in breast cancer mortality rate [12]. However, further analysis, such as gene expression analysis, would be required to verify if these genes are associated with the difference in breast cancer mortality rate.

Multiple mutation studies have consistently found that African American breast cancer patients have a higher *TP53* mutation frequency and a lower *PIK3CA* mutation frequency than White breast cancer patients [15-17]. Gene *TP53* is a tumor suppressor gene that performs essential functions in the cellular response to various stresses [18]. The role of *TP53* in cancer is well known; in animal models, it was found that mutated *TP53* almost always leads to cancer development [18]. Reinstating the function of *TP53* resulted in the regression of the tumor caused by the mutated *TP53* gene [18]. *PIK3CA* is part of a group of lipid kinases that regulate proliferation signaling pathways, and mutations in *PIK3CA* were found to increase cell invasion and metastasis [19].

Based on previous research, some genes differentially mutated between White and African American breast cancer patients are related to breast cancer. Therefore, there is a distinct possibility that mutations may have some influence on breast cancer racial disparity. Thus, because of previous studies [12], this research hypothesizes that a gene with a differential mutation analysis between two races may contribute to breast cancer racial disparity.

Combining gene expression and mutation data

Despite the studies that investigated the racial disparity using mutations or gene expression, no studies have attempted to identify genes associated with racial disparity by considering both mutations and gene expression simultaneously. Furthermore, no studies that have investigated this racial disparity have analyzed datasets that only consist of deceased breast cancer patients, and since the difference in mortality rate is being investigated, analyzing only deceased breast cancer patients is an interesting perspective that may convey some insightful results. However, since the cause of death for each patient is unknown, we also employed the All of Us database (which does not consider vital status) in our mutation analysis to ensure accuracy.

This study hypothesizes that considering both differential mutation analysis and gene expression analysis together may lead to results that not only provide more comprehensive insight into the genetics of breast cancer racial disparity but also identify possible gene targets for treatment or prevention, which may lead to more effective breast cancer treatment for African Americans and/or White people. Therefore, this study aims to identify possible genes associated with the racial disparity through mutation analysis, confirm the results from mutation analysis via gene expression analysis, and ultimately reveal possible gene targets for the treatment or prevention of breast cancer for African American and/or White people.

## Materials and methods

Data collection

Two databases were accessed. The first is TCGA (https://www.cancer.gov/tcga), a database that has sequenced and characterized over 20,000 samples spanning 33 cancer types. TCGA is maintained by the National Institutes of Health, and it focuses on collecting primary untreated tumors that were snap-frozen upon collection. Specifically, this study employed a dataset from TCGA called TCGA-BRCA. This dataset (n=1,098) was chosen because it states the vital status of the individual, which is important since this research investigated the disparity in mortality rates. For this dataset, only deceased Caucasian and African American breast cancer patients were considered. Therefore, only 142 of the 1,098 cases are evaluated. To compensate for this lack of data, this research also used the All of Us database (https://www.researchallofus.org), which is a database that emphasizes the collection of genetic data from minorities, such as African Americans. The All of Us database, which is supported by the National Institutes of Health, includes whole genome sequencing from participant blood, saliva, and/or urine samples. Using this database, many more patients were gathered (4,722 White and 4,026 African Americans) [20].

Somatic mutation analysis of TCGA

**Figure 1:**
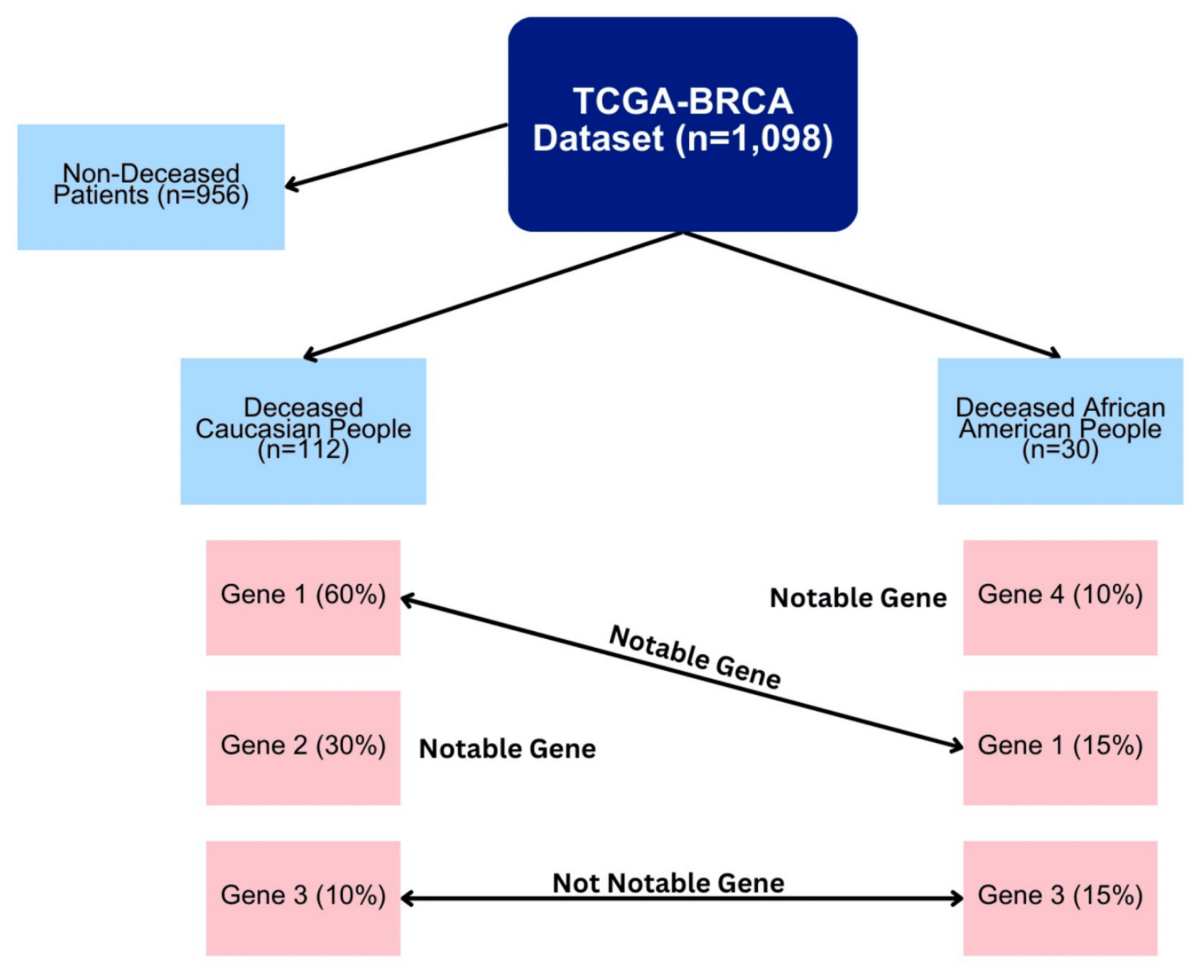
Workflow of TCGA somatic mutation analysis In this hypothetical example, the difference in relative mutation frequency for Gene 1 (60% versus 15%) between races is statistically significant (p<0.01), so it is classified as a notable gene. Gene 4 and Gene 2 are only frequently mutated in African Americans or White people, respectively, so it is also classified as a notable gene. However, Gene 3 is not notable because it is frequently mutated in both races, with similar relative mutation frequencies that are not statistically significant. TCGA: The Cancer Genome Atlas

To begin somatic mutation analysis, this study split the TCGA-BRCA dataset from TCGA into two different parts. One part consists of deceased White people (n=112), and the other consists of deceased African American people (n=30). Then, the genes with the greatest number of mutations in each of the two parts were identified. Although previous research [12] assumed that a mutation frequency greater than 5% suggests a high prevalence gene, the small size of the TCGA-BRCA dataset justifies a stricter parameter. Therefore, this study arbitrarily uses the parameter that a gene of interest must be mutated in at least 10% of the patients in that race (African American or White) to be considered a frequently mutated gene in that group. Then, the genes with the most mutations in each of the two groups were compared with each other to identify differences between the two groups. As illustrated in Figure 1, if one group had a frequently mutated gene that was not in the other group, then that gene was listed as potentially associated with the racial disparity. Additionally, if a frequently mutated gene was in both groups but was mutated far more in one group than in the other, then that gene was also listed as a potential contributor to the racial disparity. We then performed germline mutation analysis on the identified genes using the All of Us database.

All of Us database germline mutation analysis

The data from All of Us was split into two different parts: African American breast cancer patients (n=4,026) and White breast cancer patients (n=4,722). Note that the All of Us database does not provide the vital status of each of the patients. Owing to the larger sample size, the mutation frequency of the genes is significantly lower. Furthermore, the identified genes are typically mutated in at least one sample from both racial groups because of the high number of patients. Therefore, for All of Us, the crucial factor in determining possible genes associated with the racial disparity was the difference in the mutation frequency of a gene between races. As a result, the parameters for mutation analysis in the All of Us database were adjusted to require that the difference in mutation frequency between races exceeded approximately 0.5%. Applying this parameter to filter genes identified from TCGA mutation analysis results in only a handful of genes remaining. These select genes may be linked to the observed racial disparity and are now referred to as "candidate genes."

Gene expression analysis

Next, the candidate genes identified from germline and somatic mutation analysis were considered in the context of gene expression. A bioinformatics tool called TNMPlotter (https://tnmplot.com/analysis) was utilized, which can compare the gene expression of a specific gene between tumor and normal samples in breast cancer [21]. TNMPlotter uses data from either gene arrays from the Gene Expression Omnibus of the National Center for Biotechnology Information or RNA-seq from TCGA, Therapeutically Applicable Research to Generate Effective Treatments, and The Genotype-Tissue Expression repositories [21]. If the gene expression of a gene is higher in the tumor sample than in the normal sample, then the gene is overexpressed in breast cancer. If the opposite is true, then the gene is underexpressed.

**Figure 2:**
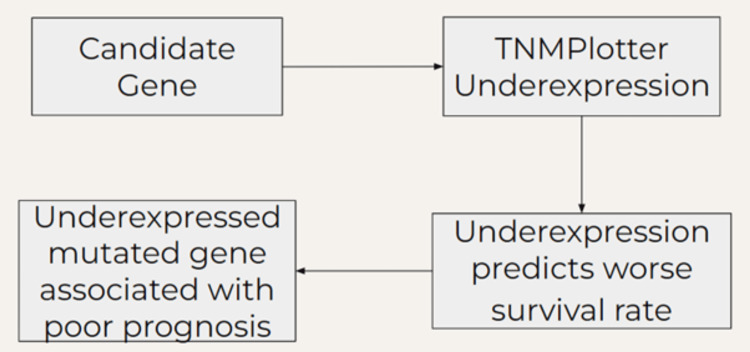
Workflow of gene expression analysis If a candidate gene is found to be underexpressed in breast cancer and its underexpression is statistically significantly (p<0.05) associated with a worse survival rate, then the underexpressed mutated gene is associated with a poor prognosis, suggesting its association with this racial disparity.

After identifying whether each candidate gene is overexpressed or underexpressed in breast cancer, this research investigated how alterations in gene expression impact the survival rates of breast cancer patients. To do so, another bioinformatics tool called the Kaplan-Meier plotter was employed [22]. The Kaplan-Meier plotter has been previously used to analyze the underexpression and overexpression of a gene and relate it to breast cancer patient survival rate [23,24]. As shown in Figure 2, if a candidate gene is found by TNMPlotter to be underexpressed and if the Kaplan-Meier plotter presents the underexpressed candidate gene variant to be associated with a lower survival rate than the overexpressed counterpart, then it can be concluded that the presence of the underexpressed mutated candidate gene is associated with poorer prognosis. If a candidate gene found to be mutated exclusively in African American breast cancer patients is found to be negatively associated with breast cancer survival rates through gene expression, then it is probable that this gene contributes to the racial difference in breast cancer mortality rate.

Overall, using this approach, analysis of TCGA and All of Us databases enabled the identification of mutated candidate genes that may play a significant role in the racial disparity observed between African American and White populations in the context of breast cancer mortality rates. Then, after considering mutations, candidate gene expression was also analyzed to assess whether they suggest an association with the racial disparity between African American and White populations. This multi-leveled method identified genes that are linked to this racial disparity through both mutation and gene expression.

## Results

Gene identification from TCGA

First, the genes that were mutated in at least 10% of one or both races were identified. For the deceased White patients (n=112), eight genes were found to be mutated in at least 10% of the patients (Table 1). For the deceased African American patients (n=30), the same procedure was executed, procuring 13 genes that were mutated in at least 10% of the patients (Table 2). A comparison of Table 1 and Table 2 reveals that while some genes are common between the two groups, others are exclusive to only one of the two groups. Additionally, although *TP53* is a common gene between the two groups, the relative mutation frequency difference (35.00% versus 54.17%) in *TP53* between the two groups is statistically significant (p<0.05). Genes that were exclusive to one group or had a statistically significant mutation frequency difference between the two groups were considered for the All of Us database mutation analysis. These genes are *LAMA1*, *MED12*, *COL12A1*, *RYR3*, *TUT7*, *SLC9A8*, *ERCC6*, *MUC16*, *KMT2C*, *SPTA1*, and *TP53*.

**Table 1:** Genes identified from TCGA White patients (n=112) These are the genes that have a relative mutation frequency rate of at least 10% in White breast cancer patients. TCGA: The Cancer Genome Atlas

Genes	Mutation frequency
TP53	35.00%
PIK3CA	32.00%
TTN	30.00%
MUC16	18.00%
KMT2C	17.00%
GATA3	13.00%
CDH1	11.00%
SPTA1	10.00%

**Table 2:** Genes identified from TCGA African American patients (n=30) These are the genes that have a relative mutation frequency rate of at least 10% in African American breast cancer patients. TCGA: The Cancer Genome Atlas

Genes	Mutation frequency
TP53	54.17%
PIK3CA	33.33%
TTN	25.00%
GATA3	16.67%
MUC17	16.67%
LAMA1	12.50%
MED12	12.50%
COL12A1	12.50%
CDH1	12.50%
RYR3	12.50%
TUT7	12.50%
SLC9A8	12.50%
ERCC6	12.50%

Candidate gene identification from the All of Us database

**Figure 3:**
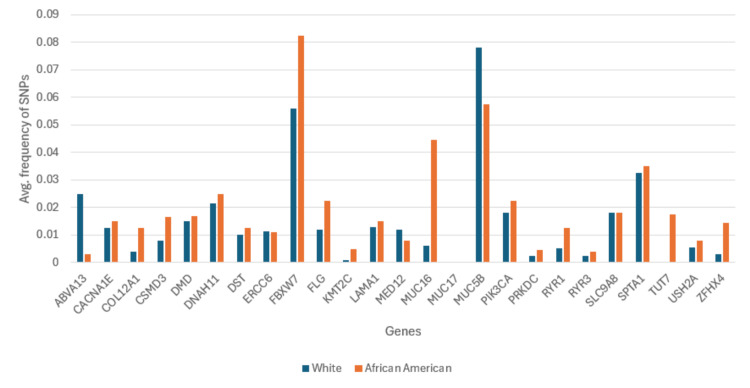
Overall SNP gene frequencies of White and African American breast cancer patients from All of Us *FBXW7* has a lower overall SNP gene frequency in White people than African Americans, and *MUC5B* has a higher overall SNP gene frequency in White people than African Americans. These disparities were found to be statistically significant (p<0.01), so *FBXW7* and *MUC5B* were considered as candidate genes. SNP: single nucleotide polymorphism

The genes identified from TCGA were then further analyzed in the All of Us database to identify candidate genes (Figure 3). Among the genes identified from TCGA, the All of Us database analysis confirmed the following candidate genes: *COL12A1*, *TUT7*, and *MUC16*. However, a few genes not identified from The Cancer Genome Atlas were found in the All of Us database to have a statistically significant (p<0.01) mutation frequency difference between the two races, *MUC5B* and *FBXW7*. Because of the statistically significant single nucleotide polymorphism (SNP) frequency disparity, these two genes were also added to the list of candidate genes. Therefore, the list of candidate genes identified for this study is as follows: *COL12A1*, *TUT7*, *MUC16*, *MUC5B*, and *FBXW7*.

Gene expression analysis

**Figure 4:**
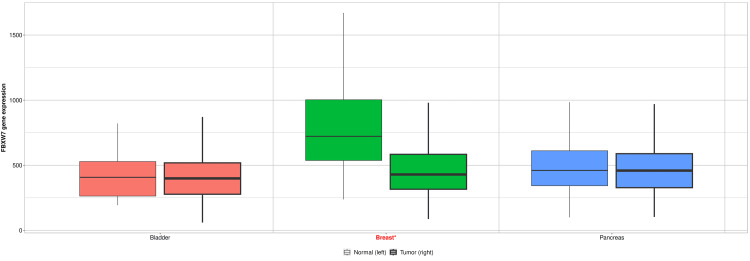
Differential gene expression of FBXW7 between normal and tumor breast cancer patients, among others Statistically significant differences (p<0.05) from the Mann-Whitney U test are marked in red. *FBXW7* is typically underexpressed in breast cancer patients.

**Figure 5:**
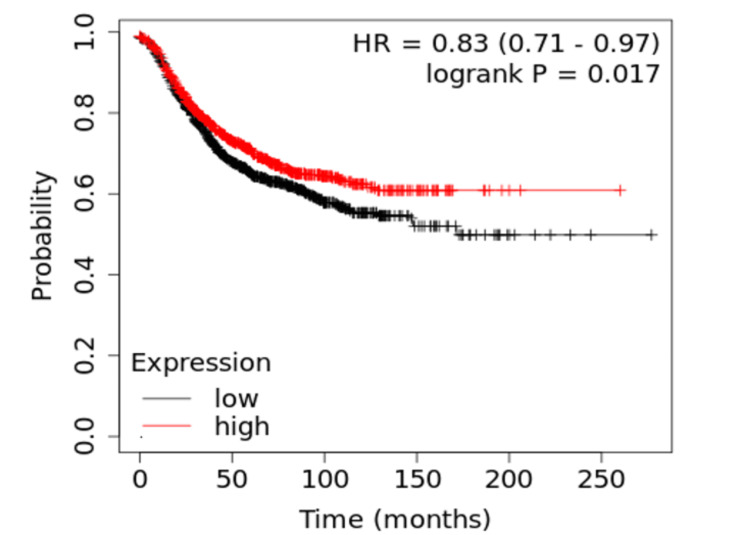
Kaplan-Meier curve for FBXW7 suggests that its low expression leads to higher mortality (p=0.017, n=2,032) The underexpressed *FBXW7 *gene was found to be statistically significantly (p<0.05) associated with a lower survival rate in breast cancer patients.

The five candidate genes identified were then analyzed for their gene expression. Of the five candidate genes, the only gene that was found to be negatively associated with the survival rate of breast cancer patients through gene expression was *FBXW7*, suggesting that this gene may be highly associated with this breast cancer racial disparity. As shown in Figure 3, SNPs in the gene *FBXW7* are more prevalent in African American patients. Additionally, Figure 4 illustrates how the gene *FBXW7* is underexpressed in breast cancer tumors (p<0.01). The statistical significance of the underexpression of *FBXW7* was calculated using the Mann-Whitney U test. Finally, the statistically significant (p<0.05) Kaplan-Meier curve in Figure 5 demonstrates that low gene expression of *FBXW7* is associated with a lower survival rate than high *FBXW7* expression in breast cancer. The other candidate genes were not found to be associated with the racial disparity between White and African American breast cancer patients in terms of gene expression.

## Discussion

Data interpretation

Gene *FBXW7* is more mutated in African American breast cancer patients, and since low *FBXW7* expression is correlated with a lower survival rate, it is reasonable to conclude that the gene *FBXW7* may be associated with the racial disparity between White and African American breast cancer patients. Moreover, the disproportionate mutation frequency of the gene *FBXW7* in African American breast cancer patients may also be a possible cause of this racial disparity. However, further research is needed to prove causality.

*FBXW7* functions as a substrate recognition component of ubiquitin ligase, playing a key role in the ubiquitin-proteasome system by targeting critical oncogenic proteins for destruction [25]. This process is essential for regulating cell differentiation, apoptosis, and metabolism [25]. Reduced *FBXW7* expression leads to abnormal stabilization of its substrates, and mutations or deletions in the *FBXW7* gene have been linked to breast cancer progression and chemoresistance [25].

The genes identified by TCGA represent the unique genetic characteristics of African Americans and White people in breast cancer. Since all the genes are either mutated in only one of the races or are disproportionately mutated between races, these genes may be associated with the racial disparity between African American and White breast cancer patients. Due to the low number of patients, however, the genes identified from TCGA were confirmed via the All of Us database to identify "candidate genes," and these candidate genes underwent gene expression analysis to identify the gene *FBXW7*. Gene *FBXW7* is a crucial part of the proteasome (protein destruction) system and is in charge of the destruction of important oncogenic (cancer-causing) proteins [25]. Since gene *FBXW7* was found to be statistically significant (p<0.05) from both mutation and gene expression analysis, it* *may likely be associated with the difference in mortality rates between White and African American breast cancer patients.

Validation of the association of *FBXW7* with breast cancer racial disparity

These results are supported by previous research, which found that low levels (gene expression) of *FBXW7* are reportedly correlated with breast cancer progression and chemoresistance [25-28]. Moreover, mutations in the gene *FBXW7* were previously shown to be associated with African American ancestry [29]. Therefore, when both mutations and gene expression are considered, gene *FBXW7* is the gene that is most likely associated with the racial disparity in breast cancer. Identifying the gene *FBXW7*, among other candidate genes, as a possible contributor to the breast cancer racial disparity is important because it leads to a greater understanding of the processes involved in the pathogenesis of breast cancer. This study not only provides insight into how tumor biological processes differ between deceased White and African American breast cancer patients but may also offer a better understanding of the causes of this survival disparity.

Candidate gene association with racial disparity

Despite the clinical significance of the *FBXW7* gene, it is important not to disregard the other genes identified in this research, especially the "candidate genes." Although these genes were not found to be significant through gene expression analysis, they were found to be potentially important from the mutation analysis. Therefore, the candidate genes may also be associated with the racial disparity in breast cancer patients. For example, a previous study discovered that *COL12A1*, a candidate gene, was more mutated in African American than in European American breast cancer patients [30]. These results correlate with those of TCGA and All of Us analysis, indicating that the *COL12A1* gene is indeed an aspect that differs between these two races. Moreover, survival analysis via Kaplan-Meier plotter done by other researchers found that gene *COL12A1* was negatively associated with the prognosis of breast cancer patients [26]. Because mutation of this gene is more prevalent in African American breast cancer patients, it is reasonable to suggest that the *COL12A1* gene may be associated with the increased mortality in African American breast cancer patients.

Limitations

This research has some potential limitations. While The Cancer Genome Atlas dataset considers only deceased African American and White breast cancer patients, the cause of death is unknown. Therefore, there may be other possible genes in the All of Us and the BRCA-TCGA datasets associated with the racial disparity between African American and White breast cancer patients that have not been identified.

Another major limitation is that this is a secondary data analysis. Furthermore, the same samples were not used for both genomic and transcriptomic analyses, and there was no secondary cohort to confirm the results. Additionally, since this bioinformatics study is not a controlled experiment, causality cannot be determined between *FBXW7* and this racial disparity.

## Conclusions

In this study, multiple candidate genes, especially *FBXW7*, were identified as possibly being associated with the racial disparity of breast cancer between African American and Caucasian patients. Additionally, the introduction of a multi-level method combining previous methods has the potential to guide further research into breast cancer and other cancers.

No laboratory procedures were performed to verify the identified candidate genes. Moreover, the lack of a controlled experiment prevents causality from being determined between these candidate genes and this racial disparity. Future work will emphasize performing a controlled experiment in a laboratory to both verify the analysis and determine causality. Furthermore, if the identified candidate genes are proven to cause the racial disparity between African American and Caucasian breast cancer patients, then clinical trials to target these genes for breast cancer treatment may also be possible.
